# Supplementary material for: The therapeutic alliance between study participants and intervention facilitators is associated with acute effects and clinical outcomes in a psilocybin-assisted therapy trial for major depressive disorder
Source: PLoS One. 2024 Mar 14;19(3):e0300501. doi: 10.1371/journal.pone.0300501 (PMC10939230; doi:10.1371/journal.pone.0300501)
Supplement: Attachment — Submitted filename: Responses to Reviewers 1.16.24.docx [file pone.0300501.s001.docx]

Thank you for the opportunity to revise and resubmit our manuscript. Attached please find the revised version of our manuscript titled “The therapeutic alliance between study participants and intervention facilitators is associated with acute effects and clinical outcomes in a psilocybin-assisted therapy trial for Major Depressive Disorder”. Below you will find the editor and reviewer’s comments and how we addressed each of their concerns and suggestions. We believe these revisions have significantly improved this manuscript, and we look forward to hearing back regarding the next steps of the review process.

**Reviewer #1:**

This paper is delightful. As the authors emphasize, the work is essential, fills a relevant gap, and addresses a key issue empirically in ways few other studies have. The Introduction motivates the analysis superbly. The consistent reporting of effect sizes is most welcome. The Discussion underscores the essential take-home messages well while touching on the necessary limitations of the current data. I’m trying to take the perspective of the folks who recently asserted, in so many words, that we really just need to keep folks safe during the acute effects and that’ll do the trick. I think that one statistical issue will be critical. I’ve identified a few points that might generate more criticism than the authors want as well.

1. Readers are bound to wonder if a handful of outliers are driving the whole effect. I don’t need a Florence Nightingale Rose or Violin Plot, but the authors should report the skew for anything where they use a Pearson’s or t-test. Anything over an absolute value of 1 will probably need a transformation or a non-parametric alternative or some other strategy to sidestep the issue. (With an N this small, the statistical tests for violations of normality will never reach significance, but they’re ass backwards anyway.) Detailed rationale and potential interventions or transformations appear in:
   Osborne JW. Best practices in data cleaning: A complete guide to everything you need to do before and after collecting your data. Sage publications; 2012 Jan 10.

   I would guess that anchoring at 1 and taking a log or square-root would not seem to abstruse for most readers.

**Author Response:** We thank the reviewer for this suggestion. Given the evidence that shows that Pearson correlations and t-tests are robust against violations of the normal distribution of scores and given that this study is a secondary data analysis from one very small clinical trial which is meant to be exploratory and hypothesis-generating (and not a definitive answer to this research question), we have chosen not to change our analytic method. However, we added details about this rationale to the methods section (lines 186-190) and we added this as a limitation of this study in the discussion section (lines 865-876).

2. The writing is completely on par with the quality in our field—a low bar. If the authors would consider eschewing passive constructions and grammatical expletives, readability would improve. The word “this” without a definite antecedent, also interrupts the flow. I am guessing that the published version will generate more attention with some tightening. These steps alone would make the Abstract more accessible and provide space to show more details or enthusiasm.

**Author Response:** We appreciate the stylistic guidance and agree that readability could be improved. As such, we have attempted to remove passive constructions and grammatical expletives throughout the manuscript and to cut words for clarity and to improve flow where possible. In service of this goal, we also replaced the term “psychedelic-assisted psychotherapy” with PAT throughout the manuscript. We also included those recommended edits in Paragraph 7 of the Discussion (lines 806-816), as we believe it significantly enhances readability.

Introduction

3. The single-item indicator is bound to generate some concern and skepticism. The authors might want to mention this paper or comparable ones supporting the validity of single-item measures.

Dollinger, S. J., & Malmquist, D. (2009). Reliability and validity of single-item self-reports: with special relevance to college students' alcohol use, religiosity, study, and social life. The Journal of General Psychology, 136(3), 231-242.

**Author Response:** We agree that the single-item indicator is worth addressing in more detail. We included an explanation/justification in both the Methods (Lines 179-181) and the Limitations section (Lines 825-845) using the reference you provided above. We also added two additional references (refs. 74, 75) highlighting recently validated (since this study) insight scales for psychedelic therapy.

4. The authors might want to mention these publications, some of which might not have appeared at the time of submission, for the sake of comprehensiveness.

Gramling, R., Bennett, E., Curtis, K., Richards, W., Rizzo, D. M., Arnoldy, F., ... & Agrawal, M. (2023). Developing a Direct Observation Measure of Therapeutic Connection in Psilocybin-Assisted Therapy: A Feasibility Study. Journal of Palliative Medicine.

As the title emphasizes, this paper points out a way to sidestep one of the shortcomings of the current data.

Kamilar-Britt P, Gordis EB, Earleywine M. The Therapeutic Alliance in Psychedelic-Assisted Psychotherapy: A Novel Target for Research and Interventions. Psychedelic Medicine. 2023 Aug 18.

This paper also shows the need for multiple raters on the alliance measures.

**Author Response:** Thank you for bringing our attention to these highly relevant recent publications. We felt that they were most appropriately incorporated into the Discussion and Limitations sections. We added the Gramling et al. 2023 citation in our discussion of measuring and defining the therapeutic alliance in psychedelic assisted therapy (Lines 809-814). We added the Kamilar-Britt et al. 2023 paper in the Limitations section (Lines 855-857), suggesting future studies incorporate multiple raters (including third-party).

5. The writing is completely comparable to published work in our field—a very low bar. Changing passive constructions to active voice and eliminating grammatical expletives would improve readability.

**Author Response:** This has been incorporated throughout as mentioned above.

Methods

6. McDonald’s Omega has become the standard on internal consistency now, in part because the approach requires fewer assumptions. I’m pretty sure SPSS has the option in a straightforward way. Details appear in:

   Hayes AF, Coutts JJ. Use omega rather than Cronbach’s alpha for estimating reliability. But…. Communication Methods and Measures. 2020 Jan 2;14(1):1-24.

   These authors walk through the issue related to assumptions of tau-equivalence, not that the current paper needs those details.

**Author Response:** Thank you for this suggestion. However, this statistic has not become the standard in our discipline, and we believe that the alpha value is a better way to communicate the internal consistency of our scales.

Results

7. I’m not enjoying the “small,” “medium,” and “large” designations with the effect sizes in the tables. Cohen would admit that he literally pulled the distinctions from thin air and the significance designations certainly help get the point across.

**Author Response:**  Thank you for your comment. Although, possibly arbitrary, the ranking of effect sizes is still widely accepted in the field and offers useful context to readers who are unfamiliar with statistics. That said, we agree that including these distinctions within the table is unnecessary and distracting. Therefore, we have removed them from the table and maintained the footnote to aid in interpretation.

Discussion

8. I think the expression “reverse causality” is ill-advised here. More readers will grow confused than entertained or enlightened

**Author Response:** We removed this term in favor of a more clear and concise statement (line 697-698): “Our findings also strongly suggest that the acute psychedelic experience directly enhances the therapeutic alliance.”

Reviewer #3:

Comment: The authors provide a nice analysis of human depression scores that emphasizes a relationship between environmental factors (therapeutic alliance) and drug effects (psilocybin-assisted therapy) in clinical population. My only concerns are the small sample size and the reliance on parametric statistical analyses to assess meaningful relationships:

1. I suggest bolstering the current piece by providing more details to the Methods Section. Specifically, the design of this study, the rationale for the number of subjects selected, and the tools/software used to generate planned analyses should include more details.

**Author Response:** We have added details to the methods section, including the design of the study and the rationale for choosing the sample size (lines 124-130). We have provided the software that we used (SPSS) in the analysis description. There were no other tools/software used in this analysis.

2. The authors should carefully consider alternatives to the current use of parametric analyses where the n is small and the range of values is large.

**Author Response:** We have addressed a similar concern provided by reviewer #1. Given the evidence that shows that Pearson correlations and t-tests are robust against violations of the normal distribution of scores and given that this study is a secondary data analysis from one very small clinical trial which is meant to be exploratory and hypothesis-generating (and not a definitive answer to this research question), we have chosen not to change our analytic method. However, we added details about this rationale to the methods section and we added this as a limitation of this study in the discussion section.
